# Everyday Language Exposure Shapes Prediction of Specific Words in Listening Comprehension: A Visual World Eye-Tracking Study

**DOI:** 10.3389/fpsyg.2021.607474

**Published:** 2021-02-09

**Authors:** Aine Ito, Hiromu Sakai

**Affiliations:** ^1^Department of German Studies and Linguistics, Humboldt-Universität zu Berlin, Berlin, Germany; ^2^Faculty of Science and Engineering, Waseda University, Tokyo, Japan

**Keywords:** language prediction, listening comprehension, orthographic processing, eye-tracking, visual world paradigm

## Abstract

We investigated the effects of everyday language exposure on the prediction of orthographic and phonological forms of a highly predictable word during listening comprehension. Native Japanese speakers in Tokyo (Experiment 1) and Berlin (Experiment 2) listened to sentences that contained a predictable word and viewed four objects. The critical object represented the target word (e.g., 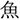/sakana/; *fish*), an orthographic competitor (e.g., 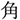/tuno/; *horn*), a phonological competitor (e.g., 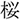/sakura/; *cherry blossom*), or an unrelated word (e.g., 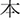/hon/; *book*). The three other objects were distractors. The Tokyo group fixated the target and the orthographic competitor over the unrelated objects before the target word was mentioned, suggesting that they pre-activated the orthographic form of the target word. The Berlin group showed a weaker bias toward the target than the Tokyo group, and they showed a tendency to fixate the orthographic competitor only when the orthographic similarity was very high. Thus, prediction effects were weaker in the Berlin group than in the Tokyo group. We found no evidence for the prediction of phonological information. The obtained group differences support probabilistic models of prediction, which regard the built-up language experience as a basis of prediction.

## Introduction

During language comprehension, people sometimes predict a word that is likely to come up and pre-activate representations of the predictable word before it is mentioned (Kamide, 2008; Kuperberg and Jaeger, 2016; Pickering and Gambi, 2018 for reviews). Prediction is likely to play an important role in online comprehension, as successful prediction can facilitate the processing of predicted information. However, there are individual differences in prediction, so people are likely to benefit from prediction to a different degree. For example, language proficiency measures such as vocabulary size and verbal fluency have been found to mediate language prediction (Rommers et al., 2015; Hintz et al., 2017). However, it is unclear how everyday exposure to the language affects prediction among native speakers. Under the probabilistic models of language prediction (e.g., Kuperberg and Jaeger, 2016), predictions are generated on the basis of statistical probabilities (i.e., the likelihood of a certain input to occur in the given context). Thus, prediction may be stronger in those who are exposed to the language more often than those who are exposed to it less often. The current study investigates the effects of everyday language exposure on the prediction of orthographic and phonological form by comparing native Japanese speakers who were resident in Japan (Tokyo) and Germany (Berlin).

### Effects of Language Exposure on Language Processing

Research on bilingual children suggests that both the quantity and quality of language exposure has a significant impact on their language acquisition (Unsworth, 2016, for a review). Language exposure at both early and late stages of learning seems to predict people’s language skills (Tao et al., 2019). According to usage-based theories of language acquisition, frequency of exposure is a key to language acquisition because people learn the rules of a language via an analysis of the distributional characteristics of the language input (Ellis, 2002). However, is language exposure also crucial for maintaining the efficiency of using a native language? Linck et al. (2009) compared adult native English speakers who were learning Spanish in Spain or in the United States in a translation recognition task. Participants were asked whether two presented words were translation equivalents in Spanish and English (e.g., cara – face). Words that were similar in form (e.g., cara – card) slowed down the judgment in participants in the United States but not in those in Spain, suggesting that immersed Spanish learners had attenuated access to English. Thus, exposure to a non-native language may impact native language processing in highly proficient native speakers who immigrated to another country in adulthood.

Effects of reduced language exposure may impact the efficiency of orthographic processing especially in languages that use many orthographically complex characters such as Japanese and Chinese, because people can forget an orthographic form of a word that they had acquired. In a survey on the Japanese language conducted by the Agency for Cultural Affairs in Japan in 2012, 66.5% of the respondents indicated that their ability to correctly write kanji characters (logogram in Japanese) had deteriorated due to the increased use of electronic communication methods, and 87% of them believed that the writing ability of the Japanese people had been deteriorating (retrieved from: https://www.bunka.go.jp/tokei_hakusho_shuppan/tokeichosa/kokugo_yoronchosa/pdf/h23_chosa_kekka.pdf). Koyama et al. (2008) found that visual long-term memory was a significant predictor of children’s writing skills for logographic kanji but not for hiragana/katakana (phonogram in Japanese), presumably because of the visual complexity of kanji characters. If frequent exposure to the language enhances visual memory, less frequent exposure, on the other hand, may result in less efficient processing for kanji characters.

According to word recognition models that support an interactive activation of phonology and orthography during listening and reading comprehension (Frost and Ziegler, 2007), an auditory stimulus (e.g., a spoken word) activates both phonological and corresponding orthographic representations. This bi-modal activation seems to depend on language proficiency, such that more proficient language users tend to have a stronger link between phonology and orthography (Veivo and Järvikivi, 2013; Veivo et al., 2016). It is then possible that more frequent exposure to a language strengthens the link and enhances orthographic activation during listening. However, this is not entirely clear based on the effect of proficiency because more proficient language users are likely to have more frequent exposure to the language.

### Evidence for Prediction

Many studies have found that people can predict upcoming information during language comprehension. For example, in a visual world eye-tracking study by Altmann and Kamide (1999), native English speakers listened to sentences such as “*The boy will eat the cake*.” or “*The boy will move the cake*.” while viewing a scene depicting a boy, a cake, and some inedible objects (e.g., toys). Participants were more likely to look at the picture of the cake when they heard “*eat*” compared to when they heard “*move*,” suggesting that they used the semantic constraints of the verb to predict a feature of an upcoming object (e.g., edible). This finding has been replicated many times (e.g., Kamide et al., 2003; Kukona et al., 2011; Ito et al., 2018a), and evidence for the prediction of semantic information has also been found in ERP (Event Related Potentials) studies (e.g., Federmeier and Kutas, 1999; Boudewyn et al., 2015; Wlotko and Federmeier, 2015; Ito et al., 2016).

When the sentence context is highly predictive, people can predict information about a specific word that is likely to occur. For example, Ito et al. (2018b) found that people predict the phonological form of a highly predictable word. In their study, native English speakers listened to sentences that contained a predictable word (e.g., “*The tourists expected rain when the sun went behind the cloud*, …”) and viewed four objects. One of the objects represented the target word (e.g., *cloud*), a phonological competitor which shared initial phonemes with the predictable word (e.g., *clown*), or an unrelated word (e.g., *globe*). Participants fixated both the target object and the phonological competitor over the unrelated object before the target word was mentioned, demonstrating prediction of phonological form of the target word. Kukona (2020) replicated these findings and further found evidence that lexical association (e.g., priming from *rain* to *cloud*) plays a role in prediction.

ERP studies have also found evidence that people predict a specific phonological or orthographic word form of a highly predictable word (e.g., Laszlo and Federmeier, 2009; Kim and Lai, 2012; Ito et al., 2016). For example, Laszlo and Federmeier (2009) presented participants with a predictive context (e.g., “*Before lunch he has to deposit his paycheck at the*…”), which was followed by the predictable word (e.g., *bank*), an orthographically related word (e.g., *bark*), an orthographically related pseudoword (e.g., *pank*), or an orthographically related non-word (e.g., *bxnk*). In other conditions, the context was followed by an orthographically unrelated word/pseudoword/non-word. They found a larger N400 for the unexpected continuations compared to the expected continuation, suggesting facilitated processing for the expected continuation. Critically, this N400 effect was smaller in the orthographically related conditions than in the orthographically unrelated conditions, although both sets of conditions were equally ill-fitting to the context (see also Kim and Lai, 2012). This effect was found to be larger when the predictability was higher than when it is lower (Ito et al., 2016). Together, these findings suggest that people can predict a word form of the predictable word.

Probabilistic models of prediction (e.g., Kuperberg and Jaeger, 2016) explain the effects of prediction in terms of people’s probabilistic beliefs about upcoming input. People build these beliefs on the basis of their previous experience, and they can predict upcoming input most accurately and confidently when their probabilistic beliefs closely match the actual statistics of the linguistic input. When people encounter a new input, they update their beliefs in accordance with the new input. Because the exposure to a language will give comprehenders a chance to update their beliefs, the amount of regular linguistic input is likely to predict the accuracy or confidence of prediction.

### Individual Differences in Prediction

While there is evidence that people predict various types of information during comprehension, there is also evidence that not everyone predicts to the same extent (Huettig, 2015). Studies found that non-native speakers were slower or less likely to predict than native speakers (see Ito and Pickering, 2021 for a review), and native-like prediction depended on their language proficiency (Hopp, 2013). Effects of language proficiency on prediction has also been found in native speakers – both in children (Mani and Huettig, 2012, 2014) and adults (Mishra et al., 2012; Rommers et al., 2015; Hintz et al., 2017). For example, Hintz et al. (2017) investigated predictors of verb-noun association based predictions (e.g., *peel* – *apple*) using the visual world paradigm and found that participants’ predictive eye movements were modulated by their receptive vocabulary and verbal fluency (measured in a test where people name as many words as possible which fall in a certain category or which starts with a certain letter in one min). Participants who had a larger receptive vocabulary and a better verbal fluency score were more likely to fixate the predictable object over distractor objects before it was mentioned. Thus, the extent to which people predict varies even among fluent adult native speakers.

Mishra et al. (2012) tested the effects of literacy on predictive eye movements in high and low literate adults, who had 15 and two years of formal education on average, respectively. Participants listened to sentences that contained a predictive adjective in Hindi (which needs to agree in gender with the noun it modifies). High literates used semantic and syntactic information of the adjective to predict an upcoming object and looked at the target object before it was mentioned. However, low literates showed no sign of prediction. They looked at the target object only after it was mentioned. These findings are striking, as they show the importance of literacy skills for prediction even in listening comprehension. A possible account for these findings is that regular reading and writing increases general contextual knowledge, statistical knowledge (e.g., transitional probability – the likelihood of a word following or preceding another word), or general processing speed, thereby contributing to a better prediction performance in high literates compared to low literates.

Rommers et al. (2015) investigated the individual differences in prediction of the shape of upcoming objects by measuring their predictive eye movements to a predictable target object (e.g., *moon* following “*In 1969 Neil Arm-strong was the first man to set foot on the*…” in Dutch) and a shape competitor (e.g., *tomato*). Participants looked at both the target object and the shape competitor before the target word was mentioned, suggesting that they pre-activated shape information of the target word (e.g., round). The predictive eye movements to the target object were mediated by their vocabulary size and category fluency, such that participants with higher vocabulary size and better category fluency scores tended to show more predictive eye movements. On the other hand, the predictive eye movements to the shape competitor were mediated by anticipatory attention (facilitation by valid cues in a spatial cueing task), such that participants who showed stronger facilitation from valid cues tended to show more predictive eye movements. These findings suggest that the prediction of different types of information may be mediated by different factors and hence may have different underlying mechanisms.

### Dissociating Prediction of Orthographic and Phonological Forms

The current study is based on Ito (2019), who tested the prediction of phonological and orthographic forms in a visual world eye-tracking experiment using Japanese. Participants listened to sentences that contained a highly predictable word and saw four words written in kanji (logogram in Japanese; Experiment 1). One of the words was the critical word, which varied across four conditions. In the target condition, participants saw the target word (e.g., 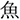/sakana/; *fish*), which was predictable from the context (see “Stimuli” section). In the orthographic condition, they saw a word that was orthographically similar to the target word (i.e., orthographic competitor: e.g., 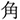/tuno/; *horn*). In the phonological condition, they saw a word that was phonologically similar to the target word (i.e., phonological competitor: e.g., 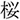/sakura/; *cherry blossom*). In the unrelated condition, they saw an unrelated word (e.g., 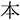/hon/; *book*).

Participants were more likely to look at the target and the orthographic competitor before the target word was mentioned. Critically, the orthographic competitor effect (i.e., the difference between the orthographic competitor and unrelated conditions) was larger when the target and orthographic competitor words were more similar versus less similar in the orthographic form, as expected, if the effect was due to the prediction of the orthographic form. However, when the same set of words were presented in hiragana (phonogram in Japanese; Experiment 2), there was no orthographic competitor effect. Since the orthographic competitor was not orthographically similar to the target word in hiragana (e.g., 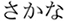/sakana/; *fish* – 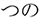/tuno/; *horn*), the findings suggest that people can pre-activate the orthographic form of a highly predictable word, but whether they do so depends on the information available in the visual context.

However, it is unclear whether people pre-activate the orthographic form of a predictable word only when the visual context provides orthographically relevant information, as there are alternative explanations for the lack of an orthographic competitor effect in the hiragana presentation. For example, the critical words used in this study are usually written in kanji, so the use of hiragana in Experiment 2 may have artificially reduced the activation of the kanji form. Another possibility is that the predictive looks to the orthographic competitor in Experiment 1 were driven by orthographic pre-activation via orthographic priming from the orthographic competitor. A related possibility is that participants may have initially mistook the orthographic competitor word for the target word. If one of these possibilities was true, we expect to find no orthographic effect in the current study, which used objects instead of printed words.

### The Current Study

The current study is a replication of Ito (2019), which use objects to rule out all the possibilities discussed above. We investigated the prediction of orthographic and phonological form in native Japanese speakers in Tokyo (Experiment 1) and Berlin (Experiment 2) to examine the effects of everyday language exposure on prediction. If people predict orthographic and phonological form of a highly predictable word, we expect more fixations on orthographic and phonological competitors over unrelated objects before the predictable word is mentioned. Additionally, if regular exposure to the target language facilitates the use of statistical probabilities (cf. Kuperberg and Jaeger, 2016), we expect prediction effects to be stronger in the Tokyo group than in the Berlin group.

We discussed in the introduction that multiple factors related to language proficiency seem to modulate prediction independently. To explore which factors affect prediction most significantly, we additionally tested the effects of language proficiency by testing correlations between the degree of predictive eye movements and language proficiency measured in verbal fluency tests and kanji reading and writing tests. Studies that found a relationship between verbal fluency and the prediction (Rommers et al., 2015; Hintz et al., 2017) took the finding to suggest that a production based mechanism is involved in language prediction (among other mechanisms), in line with production-based prediction models (e.g., Pickering and Garrod, 2013). If verbal fluency mediates prediction in general, we expect to find a relationship between verbal fluency and the prediction of orthographic and phonological form. However, it is also possible that we do not find such a relationship, as the prediction of different types of information may be modulated by different factors (Rommers et al., 2015).

The investigation of the effects of kanji reading and writing skills was motivated by Mishra et al.’s (2012) study, which found that semantic and syntactic predictions were modulated by people’s literacy skills. If literacy skills are important for prediction in general, kanji reading and writing skills may modulate the prediction of orthographic and phonological forms as well. However, our participants were all highly literate (unlike in Mishra et al.), and individual differences in literacy skills were much smaller. If literacy skills do not play a role among highly literate languages users, we may find no effect of literacy skills on prediction.

## Experiment 1

### Methods

#### Participants

Fifty-seven native Japanese speakers studying at Waseda University in Tokyo, Japan (24 males, age *M* = 21 years, range = 18–25 years) participated in Experiment 1. They had normal or corrected-to-normal vision. All participants reported Japanese to be the dominant language and they had learnt English in a school setting. The other languages participants had learnt included Chinese (15 participants), German (10), Spanish (9), French (7), Russian (5), Italian (1), Korean (1), Portuguese (1), Norwegian (1), and Ainu (1). Sixteen participants reported a regular use of English (e.g., English classes), one participant reported a regular use of Norwegian, and one participant reported a regular use of German. Nine further participants were tested but were excluded from analysis because they almost never fixated (less than 5%) on any critical object in the analyzed time window.

#### Stimuli

The auditory stimuli were identical to those used in Ito (2019), which included 20 critical sentences that contained a highly predictable target word (e.g., 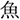 *fish*), such as in the example below.


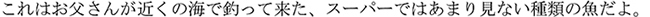


**Gloss translation:** 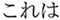 This-_*Nominative*_/
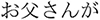
 father-_*Nominative*_/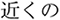 nearby/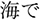 in the ocean/
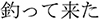
 caught/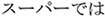 in supermarkets/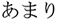 rarely/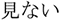 not found/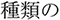 a kind of/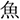 fish/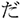-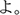
is-_*Particle*_.

“*This is a fish that my father caught in the ocean nearby, a type of which is rarely found in supermarkets.*”

The mean cloze probability for the target words was 87% (SD = 9%). The mean cloze probability including only target responses in kanji was 77% (SD = 15%). Thus, there was a preference to write the target words in kanji (rather than other Japanese scripts). The sentences were recorded by a native male Japanese speaker using a Marantz PCM recorder. The mean duration of the target words was 261 ms. The experiment used an additional 20 filler sentences that were not predictive toward a specific word. These sentences mentioned one of the depicted objects 75% of the time, so the sentences in the entire experiment mentioned one of the depicted objects 50% of the time.

**FIGURE 1:**
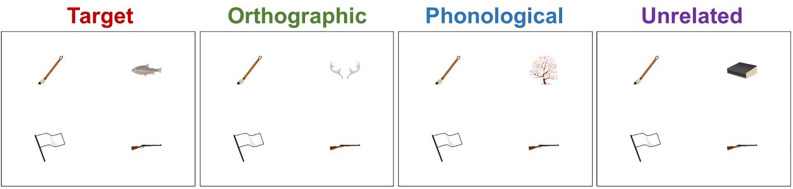
An example of the visual stimuli for each condition. The object on the top right was the critical object for this item: 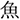/sakana/(*fish*) for the target condition, 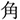/tuno/(*horn*) for the orthographic condition, 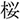/sakura/(*cherry blossom*) for the phonological condition, and 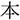/hon/(*book*) for the unrelated condition. The distractors, 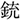/jyuu/(*gun*), 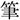/hude/(*writing brush*), and 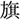/hata/(*flag*) were identical across the conditions.

The visual stimuli were created by replacing the words in Ito (2019) with objects. Three words were changed because they were hard to depict (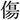/kizu/; *injury* 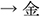/kin/; *gold*, 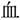/chi/; blood 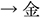/kin/; *gold*, and 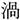/uzu/; *eddy* 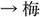/ume/; *plum*). Each display depicted four objects: one critical object and three distractor objects (Figure 1). Only the critical object differed across the four conditions. In the *target condition*, the critical object represented the predictable target word (e.g., 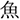/sakana/; *fish*). In the *orthographic condition*, it represented a word that was orthographically similar to the target word in kanji but phonologically dissimilar to the target word (e.g., 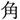/tuno/; *horn*). In the *phonological condition*, it represented a word that was phonologically similar to the target word but orthographically dissimilar to the target word in kanji (e.g., 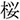/sakura/; *cherry blossom*). The phonological condition always shared one-mora with the target condition, because phonological priming in Japanese generally requires at least one-mora overlap (Kureta et al., 2006; Verdonschot et al., 2011). In the *unrelated condition*, the critical object represented a word that was orthographically or phonologically dissimilar to the target word (e.g., 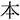/hon/; *book*). Orthographic similarity with the target word was assessed in a rating test in Ito (2019) and is shown in Table 1. In this test, native Japanese speakers (*N* = 38) rated how similar the paired words looked on a scale from 1 (= not similar at all) and 7 (= very similar).

**TABLE 1:** Kanji and lexical characteristics of critical words in each condition.

Condition	Stroke	Grade of kanji acquisition	Log kanji frequency	Mora count	Orthographic similarity
Target	7.4 (3.1)	3.5 (2.5)	4.2 (0.78)	2.1 (0.51)	—
Orthographic	7.5 (3.3)	3.5 (2.5)	4.1 (0.84)	2.1 (0.55)	3.9 (2.8–5.9)
Phonological	8.9 (4.2)	4.6 (2.4)	4.2 (0.90)	2.2 (0.41)	1.4 (1.1–1.8)
Unrelated	7.5 (4.0)	4.0 (2.5)	4.2 (0.90)	2.1 (0.61)	1.4 (1.1–1.8)

*(From the left column) The mean number of strokes of kanji, the mean grade of kanji acquisition (in which elementary-school grade kanji is taught; cf. grade 3 is for 8–9 year-olds), the mean log kanji frequency, the mean mora count, and the orthographic similarity with the target word in kanji (scale: 1–7). The SDs or range is in brackets.*

The critical objects were never presented together to make the orthographic or phonological relatedness less obvious and to prevent fixations on the competitor objects from being swamped by fixations on the target object. In all conditions except for the target condition, the critical object was implausible to be mentioned in the target word position. Care was taken to ensure that the critical word in the orthographic/phonological/unrelated conditions was not semantically related to the target word. All critical and distractor words were one-kanji character words. The characteristics of the critical words were obtained from www.kanjidatabase.com (Tamaoka et al., 2017) and are shown in Table 1. All critical words were selected from joyo kanji – 2136, a list of commonly used kanji announced by the Japanese government in 2010. There were no differences across the conditions in the number of strokes, the grade of acquisition and frequency of the kanji, or in mora counts of the word (one-way ANOVAs, *p*s > 0.4). The full list of critical sentences and object names are in the Appendix.

All critical objects were presented again on filler trials with a non-predictive sentence and distractors from another item to test whether there is any fixation bias toward a particular object (e.g., due to their visual features). We tested whether the critical object in each condition was similarly likely to be fixated when the sentence does not mention them (cf. “Results” section).

The stimuli were pseudorandomized and divided into four lists with two versions. Each list contained the same number of trials per condition and contained only one condition per item. The two versions were created by swapping the first half and the second half. The critical object appeared in each of the quadrants equally frequently. Due to the limited number of one-character words, some of the critical objects were used more than once, but they were never used in the same condition or presented in succession.

#### Procedure

Before the eye-tracking experiment, participants were familiarized with the objects. In the training phase, all objects (including the distractor objects) were presented with their name one by one, and participants were instructed to memorize them so that they could name them later. In the testing phase, participants saw only the object and named it. Incorrectly named objects were repeated until they were named correctly. The mean accuracy of naming in the first instance was 99%, suggesting that it was easy for the participants to associate the objects with their intended name.

In the eye-tracking experiment, participants were instructed to listen to the sentences via headphones and click on an object that was mentioned in the sentence or click on the background if none of the objects was mentioned. Participants’ eye movements were recorded using an EyeLink 1000 Desktop mount eye-tracker sampling at 500 Hz. Each trial began with a drift check (i.e., participants fixated at the center of the screen at the beginning of the trial). Participants then heard the sentence, and the objects appeared on the screen 1000 ms before the target word. The mouse pointer appeared in the center of the screen when the objects appeared, and it disappeared when participants clicked on an object or the background. The objects remained on the screen until 3000 ms after the sentence offset. The experiment began with four practice trials, and the main experiment was divided into two blocks. Calibration (using a five-point grid) and validation were performed before the practice session as well as before the main experiment and before the second block, if necessary. The visual scenes were presented on a monitor at a resolution of 1280 × 1024 pixels.

After the eye-tracking experiment, participants performed verbal fluency tests (letter fluency and category fluency) and kanji reading and writing tests adapted from kanken (https://www.kanken.or.jp/). We used three letters (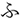/hu/, 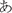/a/and 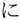/ni/) for the letter fluency test and three categories (animals, vegetables, and home appliances) for the category fluency test. In the verbal fluency tests, participants named as many words as possible that started with the given letter (letter fluency) or that belonged to the given category (category fluency). In the kanji reading test, participants were presented with 100 words written in kanji and wrote down their readings in hiragana. In the kanji writing test, they were presented with 100 words written in katakana (another phonogram in Japanese) and wrote down their kanji form. These words were embedded in a context sentence so that the meaning of the word was not ambiguous (in case there were homophones). Finally, participants filled in a questionnaire which asked how many hours they read and write (excluding typing with a computer or a smartphone) in Japanese per week, the non-native language(s) they can speak and regularly use, the length of education in a non-native language environment, and their experience of living abroad. The entire experiment took about 90 min^1^.

#### Eye-Tracking Data Coding and Analysis

We analyzed the eye movement data using mixed-effects logistic regression models including linear and quadratic time (using orthogonal polynomials) to capture both overall differences between the conditions and effects over the time-course (Mirman et al., 2008). These time terms were chosen because the model with these terms captured the observed data well. The fixations on critical objects were coded binomially (1 = fixated, 0 = not fixated) for each 50 ms bin relative to the target word onset. Fixations were regarded as falling on an object if they fell in the area of 300 × 300 pixels surrounding the object and fixations were regarded as falling on the background if they did not fall on any of the objects. No trials were excluded from the analysis, but the bins that contained only blinks were coded as NA (1.5% of the data). We initially created a model which tested the fixed effects of time (linear, quadratic) and condition (target vs. unrelated, orthographic vs. unrelated, phonological vs. unrelated) as well as an interaction of time by condition, including by-participant and by-item random intercepts and by-participant and by-item random slopes for time and condition. When this model did not converge or had a singular fit, we simplified the model by dropping the variable(s) that accounted for the least variance. The variable condition was dummy-coded with the unrelated condition as the reference condition (because it was the control condition). To capture predictive effects (i.e., effects that occurred before the target word could be processed), this model was run in the time window from 200 ms after the scene onset (= 800 ms before the target word onset) to 200 ms after the target word onset, assuming a 200 ms lag to initiate saccades (Saslow, 1967). The R script and data used for the analysis are available on OSF (https://osf.io/6q7mh/).

### Results

#### Comprehension Task

The mean accuracy for the clicking task was very high (*M* = 98%, SD = 6.7%), suggesting that participants were paying attention to the sentences.

#### Eye-Tracking Data

**FIGURE 2:**
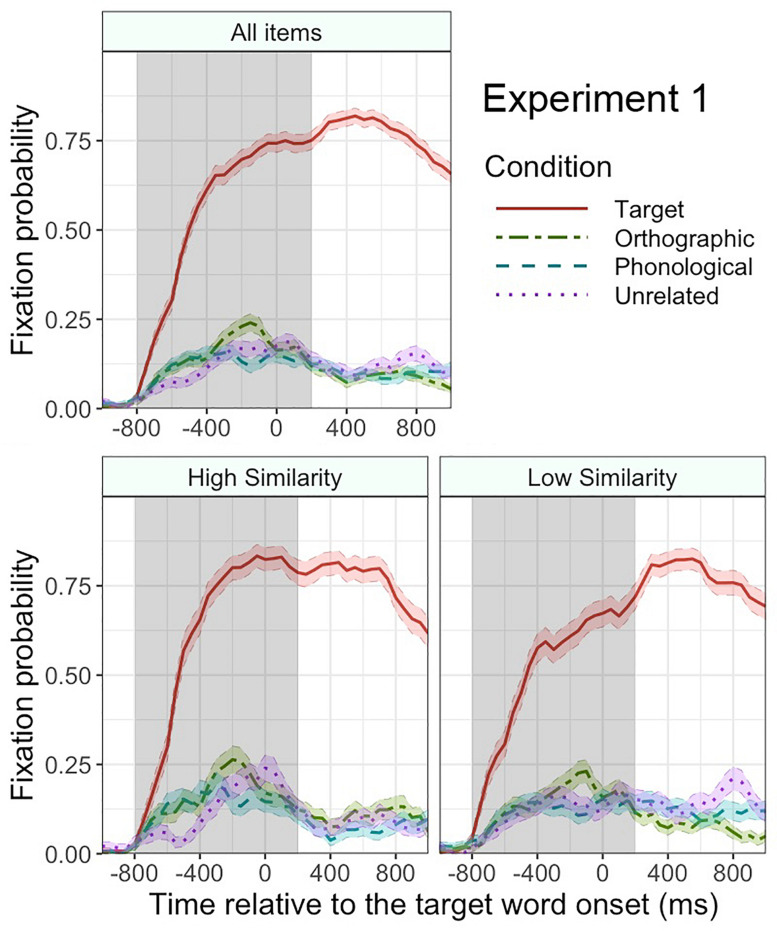
Mean fixation probabilities with standard error (shaded area around each line) for each condition in Experiment 1. The results for all items (top panel), high orthographic similarity items (bottom left panel), and low orthographic similarity items (bottom right panel). The gray shaded area indicates the analyzed time window. Time 0 indicates the onset of the target word.

Figure 2 plots the mean fixation probabilities for the critical object in each condition. In the time window from -800 ms to 200 ms relative to the target word onset, the target objects attracted more fixations overall than the unrelated objects (57.5% vs. 14.6%), β = 2.8, SE = 0.18, *z* = 15.3, *p* < 0.001. This difference increased over time, as revealed by a significant interaction of this effect with linear time, β = 2.3, SE = 0.30, *z* = 7.8, *p* < 0.001. The fixation probability for the target condition also had a clearer peak than the unrelated condition, as revealed by a significant interaction with quadratic time, β = −1.6, SE = 0.27, *z* = −6.0, *p* < 0.001. The orthographic competitors also attracted more overall fixations than the unrelated objects (17.8% vs. 14.6%), β = 0.41, SE = 0.14, *z* = 2.9, *p* = 0.003. The orthographic competitor effect interacted with linear time, β = −0.72, SE = 0.31, *z* = −2.4, *p* = 0.02, which indicates that the effect decreased over time. The interaction seems to be driven by the sharp decrease in fixation on the orthographic competitors at the end of the time window (around 0–200 ms relative to the target word onset). The orthographic competitor effect also interacted with quadratic time, β = −0.98, SE = 0.28, *z* = −3.5, *p* < 0.001, indicating a clearer peak in the fixation probability in the orthographic competitor condition than in the unrelated condition. The phonological competitors attracted more fixations than the unrelated objects (15.3% vs. 14.6%), β = 0.32, SE = 0.12, *z* = 2.6, *p* = 0.01, but we treat this effect with caution because it was not significant in a *t*-test^2^. An equivalent analysis on filler trials found no significant effect of condition, *p*s > 0.2. Thus, the predictive looks found on critical trials are unlikely to be due to certain features (e.g., visual attractiveness) of the critical objects.

Following Ito (2019), we tested whether the orthographic competitor effect was stronger when the orthographic competitors were more similar to the target words than when they were less similar. For illustration purposes, Figure 2 shows fixation plots for high orthographic similarity items and low orthographic similarity items (based on a median-split, median = 3.7). We created a model based on the model described above by including a fixed effect of orthographic similarity rating between the target words and the orthographic competitors and an interaction of condition by orthographic similarity. The variable orthographic similarity was entered as a numeric variable and was centered. This model showed a significant effect of the orthographic (vs. unrelated) condition, β = 0.50, SE = 0.19, *z* = 2.7, *p* = 0.008, but the orthographic competitor effect did not interact with orthographic similarity, *p* = 0.5. Thus, the orthographic competitor effect was not dependent on the orthographic similarity.

#### Individual Difference Analysis

**FIGURE 3:**
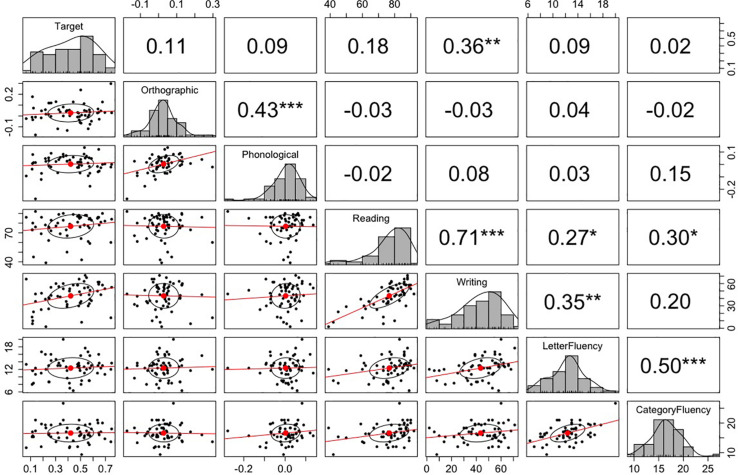
The correlation matrix among the fixation probability differences and language proficiency measures in Experiment 1. The top-right values are *r*-values (Pearson’s correlation coefficient) for the correlation tests between the variables corresponding to *x*-axis and *y*-axis. The stars next to the *r*-values indicate the significance of the correlation: *** indicates *p* < 0.001, ** indicates *p* < 0.01, and * indicates *p* < 0.05. The midline histograms show the distribution of each set of data. The bottom-left scatterplots show correlation between the variables corresponding to the *x*-axis and *y*-axis. Note: Reading/Writing = kanji reading/writing test score.

We further tested whether participants’ predictive eye movements were mediated by their language proficiency test scores. The mean scores for the letter fluency test and category fluency test were 12.4 (SD = 2.8) and 16.6 (SD = 3.0), respectively. For kanji reading and writing tests, the scores represent the proportion of correct answers. The mean scores for kanji reading and writing tests were 76.7 (SD = 12.2) and 43.5 (SD = 16.7), respectively. Each of these scores was correlated with the fixation probability differences between the target and unrelated conditions, the orthographic and unrelated conditions, and the phonological and unrelated conditions in -800–200 ms relative to the target word onset. Figure 3 plots the correlation matrix. The correlational analysis did not show any significant correlations between the predictive looks and language proficiency measures, except for the positive correlation between predictive looks to the target objects and kanji writing score, *p* = 0.006. Interestingly, there was a significant positive correlation between the fixation bias toward the orthographic competitor and the fixation bias toward the phonological competitor, *p* < 0.001 (note that the target and the orthographic competitor were not phonologically related, and the target and the phonological competitor were not orthographically related). We will come back to this point in the “General Discussion.”

### Discussion

Experiment 1 found that participants were more likely to fixate the target object over the unrelated object before the target word was mentioned, suggesting that participants predicted some information about the target word. Critically, they were also more likely to fixate the orthographic competitor over the unrelated object, suggesting that participants predicted the orthographic form of the target word. We did not find evidence for the prediction of phonological form. In the individual difference analysis, we did not find any clear relationship between predictive eye movements and language proficiency measures, except that participants with higher kanji writing scores were more likely to predictively fixate target objects over unrelated objects (However, this effect was not found in Experiment 2).

## Experiment 2

Experiment 2 asked whether regular exposure to the language affects prediction by replicating Experiment 1 in native Japanese speakers in Berlin. To quantify the participants’ language background, we asked participants to fill in a LEAP Questionnaire before the experiment (Marian et al., 2007). The questions included what languages they spoke, how much they were exposed to those languages, age of acquisition of Japanese and German, how long they had lived in Japanese-speaking and German-speaking countries, and self-rated proficiency (speaking, listening, and reading) in Japanese and German.

### Methods

#### Participants

Fifty-six native Japanese speakers who were resident in Berlin, Germany at the time of testing (10 males, age *M* = 27 years, range = 20–34 years) participated in the experiment. They had been living in a German-speaking country for 22.1 months on average (range = less than 1–120 months). The reported mean age of acquisition of Japanese was 1.3 (range = 0–7 years). All participants spoke English as a non-native language. Other languages participants spoke included German (*N* = 47), Korean (*N* = 5), Chinese (*N* = 3), Italian (*N* = 2), French (*N* = 1), Ilonggo (*N* = 1), Dutch (*N* = 1), Croatian (*N* = 1), Hungarian (*N* = 1), and Russian (*N* = 1). They reported they were exposed to Japanese 43% of the time, English 30%, and German 25%, on average. Their mean self-rated proficiency was 9.4 for Japanese and 2.9 for German (on a 10-point scale, averaged across speaking, listening, and reading). All participants had normal or corrected-to-normal vision. Eight further participants were tested but were excluded from analysis because they almost never fixated (less than 5%) on any critical object in the analyzed time window.

#### Stimuli and Procedure

The stimuli and procedure were identical to Experiment 1, except that participants filled in a LEAP Questionnaire (Marian et al., 2007) before the experiment. In the picture familiarization test, the mean accuracy of naming in the first instance was 98%, which was similarly high as in Experiment 1.

### Results

#### Comprehension Task

The mean accuracy for the clicking task was very high (*M* = 99%, SD = 1.9%), suggesting that participants were paying attention to the sentences.

#### Eye-Tracking Data

**FIGURE 4:**
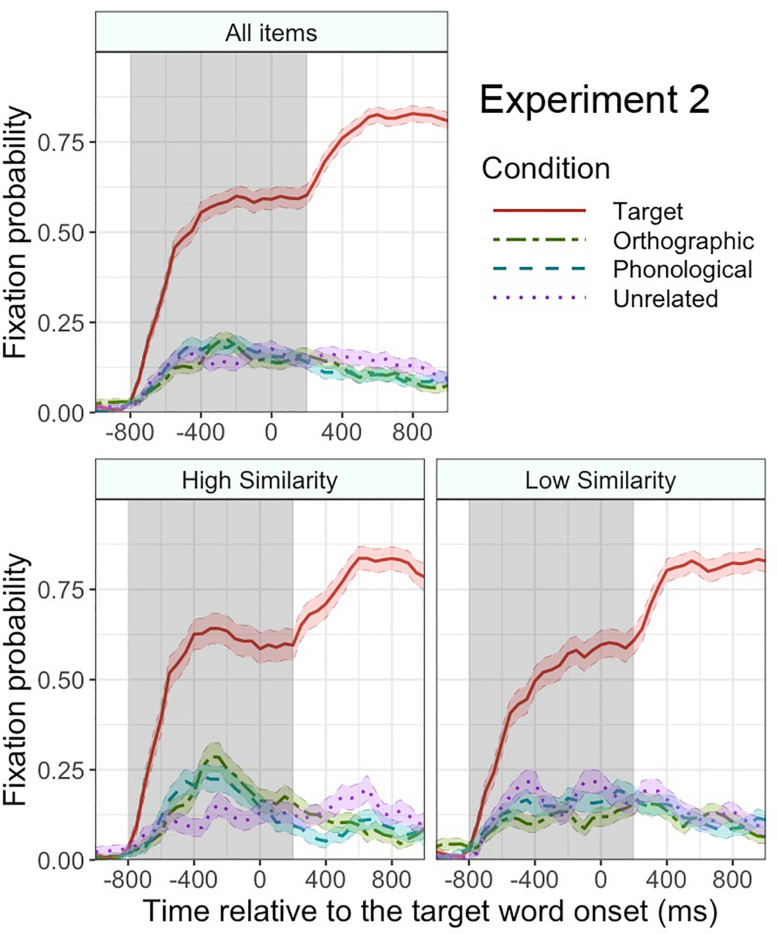
Mean fixation probabilities with standard error (shaded area around each line) for each condition in Experiment 2. The results for all items (top panel), high orthographic similarity items (bottom left panel), and low orthographic similarity items (bottom right panel). The gray shaded area indicates the analyzed time window. Time 0 indicates the onset of the target word.

Figure 4 plots the mean fixation probabilities for critical objects in each condition. Consistent with Experiment 1, the target objects attracted more fixations overall than the unrelated objects (51.0% vs. 16.0%), β = 2.1, SE = 0.26, *z* = 8.0, *p* < 0.001, in the time window from -800 to 200 ms relative to the target word onset. This difference increased over time, as revealed by a significant interaction of this effect with linear time, β = 1.7, SE = 0.26, *z* = 6.4, *p* < 0.001. The fixation probability in the target condition also had a clearer peak than that in the unrelated condition, as revealed by a significant interaction with quadratic time, β = −1.0, SE = 0.25, *z* = −4.1, *p* < 0.001. Unlike in Experiment 1, the orthographic competitors did not attract more overall fixations than the unrelated objects (15.3% vs. 16.0%), β = −0.15, SE = 0.23, *z* = −0.66, *p* = 0.5. The phonological competitors did not attract more fixations than the unrelated objects either (17.1% vs. 16.0%), β = −0.09, SE = 0.25, *z* = −0.36, *p* = 0.7. Thus, participants predictively fixated the target objects but not the orthographic competitors or phonological competitors^3^. An equivalent analysis on filler trials found no significant effect of condition, *p*s > 0.2.

Similar to Experiment 1, we tested whether the fixation bias toward the orthographic competitors was affected by the orthographic similarity between the target word and the orthographic competitor word. As can be seen in Figure 4, the overall fixation difference between the orthographic and unrelated conditions was not significant, *p* = 0.4, but there was a marginally significant interaction of orthographic (vs. unrelated) condition by orthographic similarity, β = 0.28, SE = 0.16, *z* = 1.7, *p* = 0.08, suggesting that the participants’ tendency to fixate the orthographic competitor over the unrelated object was stronger when the orthographic similarity was higher.

#### Individual Difference Analysis

**FIGURE 5:**
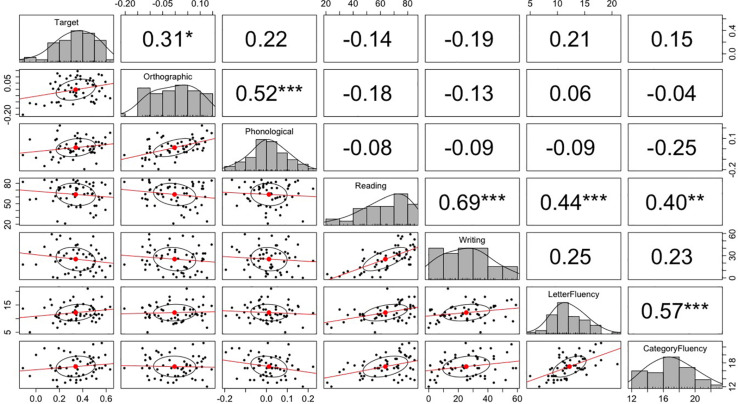
The correlation matrix among the fixation probability differences and language proficiency measures in Experiment 2. The top-right values are *r*-values (Pearson’s correlation coefficient) for the correlation tests between the variables corresponding to *x*-axis and *y*-axis. The stars next to the *r*-values indicate the significance of the correlation: *** indicates *p* < 0.001, ** indicates *p* < 0.01, and * indicates *p* < 0.05. The midline histograms show the distribution of each set of data. The bottom-left scatterplots show correlation between the variables corresponding to the *x*-axis and *y*-axis.

We tested individual differences in participants’ predictive eye movements the same as in Experiment 1. The mean scores for the letter fluency test and category fluency test were 12.2 (SD = 3.1) and 17.1 (SD = 2.7), respectively. These scores did not differ from participants in Experiment 1, *p*s > 0.3 (independent samples *t*-tests). The mean scores for kanji reading and writing were 63.7 (SD = 16.4) and 25.4 (SD = 15.9), respectively. These scores were significantly lower than those in Experiment 1, *p*s < 0.001. This is not surprising, as participants in Tokyo probably read and wrote kanji more regularly than participants in Berlin. Figure 5 plots the correlation matrix. The correlational analysis did not show any significant correlations between the predictive looks and language proficiency measures. The positive correlation between predictive looks to the target objects and kanji writing score in Experiment 1 was not replicated in Experiment 2. However, consistent with Experiment 1, Experiment 2 found a significant correlation between the fixation bias toward the orthographic competitor and the fixation bias toward the phonological competitor.

#### Between-Experiment Comparison

We tested the effect of group (Tokyo, Berlin) on predictive eye movements by including the factor group into the model that tested an interaction of condition by orthographic similarity with linear and quadratic terms. We dropped the phonological condition because neither group showed a phonological competitor effect. Thus, the model tested an interaction of condition (target vs. unrelated, orthographic vs. unrelated) by orthographic similarity by group. The factor group was deviation-coded. The model revealed more overall fixations on the target objects than the unrelated objects (54.2% vs. 15.3%), β = 2.5, SE = 0.23, *z* = 10.9, *p* < 0.001, and this effect interacted with group, β = −0.36, SE = 0.12, *z* = −2.8, *p* = 0.004, indicating that the effect was larger in the Tokyo group than in the Berlin group (target-unrelated condition difference: 42.9% vs. 35.0%). The overall difference between the orthographic condition and the unrelated condition was not significant, *p* = 0.3, but there was a significant interaction of orthographic (vs. unrelated) condition by group, β = −0.26, SE = 0.09, *z* = −2.8, *p* = 0.006, indicating that the orthographic competitor effect was also larger in the Tokyo group than in the Berlin group (orthographic competitor-unrelated condition difference: 3.2% vs. -0.7%). The model further revealed a significant interaction of orthographic (vs. unrelated) condition by orthographic similarity, β = 0.21, SE = 0.10, *z* = 2.1, *p* = 0.04. Critically, the three-way interaction of orthographic (vs. unrelated) condition by orthographic similarity by group was not significant, β = 0.03, SE = 0.04, *z* = 0.76, *p* = 0.4, suggesting that both groups showed a larger orthographic competitor effect for high (vs. low) orthographic similarity items.

### General Discussion

In two experiments, we found that participants showed a fixation bias toward the target object and the orthographic competitor relative to the unrelated object before the target word was mentioned. The predictive orthographic competitor effect suggests that people can pre-activate the orthographic form of a highly predictable word during listening comprehension. Interestingly, the Berlin group showed fewer predictive looks to the target than the Tokyo group. Additionally, the Berlin group did not show the orthographic competitor effect similar to the Tokyo group, although they showed a tendency to fixate the orthographic competitor when the orthographic competitor was highly similar to the target word. These group differences suggest that everyday language exposure affects language prediction. We found no evidence for the prediction of phonological form. The predictive looks to the target, the orthographic competitor, and the phonological competitor did not correlate with participants’ verbal fluency or kanji reading/writing scores.

These findings extend Ito (2019) and suggest that the availability of orthographic information is not a prerequisite for orthographic pre-activation to occur during listening comprehension. They further rule out the possibility that the orthographic competitor effect in Ito (2019) occurred solely because participants mistook the orthographic competitors for the target word, because the orthographic competitors were not visually similar to the target objects in the current study. The evidence for orthographic activation without any orthographic stimuli suggests that orthographic form is routinely activated during listening comprehension, providing support for interactive activation models (e.g., Frost and Ziegler, 2007). The orthographic competitor effect was relatively small, which is consistent with previous findings that a phonological or orthographic competitor effect tends to be weaker in a visual world paradigm using pictures than in a printed word visual world paradigm (Huettig and McQueen, 2007).

The results suggest that native speakers in Tokyo made stronger predictions about the target word and the orthographic form of the target word than native speakers in Berlin. This finding is consistent with probabilistic models of prediction (e.g., Kuperberg and Jaeger, 2016), which propose that people build probabilistic knowledge on the basis of their language experience and use that to predict likely upcoming input. The Berlin group reported that they were exposed to Japanese 43% of the time, so it is conceivable that they had fewer chances to update their probabilistic knowledge, and hence they might have been less accurate or less confident in their prediction. Given that the two groups showed very similar verbal fluency scores, it is unlikely that the group differences are driven by the difference in language production skills. It is also unlikely that the group differences in kanji reading and writing scores contributed to the group differences in predictive eye movements, because we did not find a clear relationship between kanji reading/writing scores and predictive eye movements in the individual difference analysis. Although we cannot rule out the possibility of other mediating factors that were not tested in this study, our study demonstrates the group differences in prediction in highly fluent native speakers.

The lack of a phonological competitor effect in our study seems inconsistent with Ito et al. (2018b), who found a predictive phonological competitor effect in a similar design with a much smaller sample size (*N* = 24) in English. A possible explanation for this inconsistency is that the phonological relatedness between the phonological competitors and the target words was not strong enough to elicit a phonological competitor effect in our study. While one-mora overlap seems sufficient to elicit a phonological priming effect in Japanese (Kureta et al., 2006; Verdonschot et al., 2011), it might not be sufficient to drive eye movements to a phonological competitor in a visual world study. Teruya and Kapatsinski (2019) used two-mora CVCV structure words in Japanese and found a phonological competitor effect when the phonological competitor word had a CVC overlap with the target word (e.g., *kamo*; duck – *kame*; turtle) but not when it only had a CV overlap (e.g., *nasu*; aubergine – *nabe*; pot). In our study, 15 out of the 20 phonological competitors had only a CV overlap. Thus, it is possible that participants pre-activated the phonological form of the predictable word, but the current manipulation did not have a power to detect it.

In both experiments, we found that participants who showed a stronger bias toward the orthographic competitors (over the unrelated objects) tended to show a stronger bias toward the phonological competitors. The orthographic competitors were not phonologically related to the target, and the phonological competitors were not orthographically related to the target (when they were written in the most preferred script – kanji). Thus, we did not expect to find this correlation. One possibility is that participants who predicted orthographic form also predicted phonological form. Participants who showed an orthographic competitor effect arguably predicted the specific word (e.g., the lexical item *fish*, rather than less detailed information such as something one can catch in the ocean), which should spread activation to the associated phonological form (Frost and Ziegler, 2007). Another possibility, which is not related to prediction, is that participants who are sensitive to the orthographic overlap are also more likely to be sensitive to the phonological overlap. Alternatively, the correlation could be related to the efficiency of activating word form from the depicted objects. Participants who were faster to retrieve the names of the objects may have been more likely to show both orthographic and phonological competitor effects.

Finally, we did not find any effect of language proficiency (letter/category fluency, kanji reading/writing scores) on predictive eye movements. The null effects contrast with studies that found individual differences on predictive eye movements (Rommers et al., 2015; Hintz et al., 2017). As we discussed in the introduction, the inconsistency may suggest that different types of prediction are mediated by different factors. Alternatively, there are differences between the current study and previous studies that could account for the inconsistency. Hintz et al. (2017) found that participants’ verbal (letter and category) fluency scores correlated with their predictive eye movements when participants had a long preview of the visual scene but not when the objects were presented only 500 ms before the target word onset. Rommers et al. (2015) presented objects 1000 ms before the sentence onset (roughly 8000 ms before the target word on average) and found that participants’ category fluency scores correlated with their predictive looks to the target. In the current study, objects were presented 1000 ms before the target word onset, so the preview might not have been long enough for the language production skills (measured in verbal fluency measures) to play a role.

Mishra et al. (2012) found effects of literacy skills on predictive eye movements in native speakers of Hindi, but the current study did not find consistent effects of kanji reading/writing scores on predictive eye movements. As we mentioned in the introduction, a clear difference between the two studies is that our participants all had formal education up to high school at the minimum, whereas the low literates tested in Mishra et al. had only two years of formal education on average. Our participants were able to read all kanji used in critical items, and the variance among the participants in kanji reading/writing scores reflect how well they knew more difficult, less frequently used kanji. We tentatively speculate that individual differences in kanji reading/writing skills beyond those needed for everyday reading/writing activities may not robustly affect prediction. However, we note that this speculation is based on the null results, and further investigations are required to better understand the effects of kanji reading/writing skills.

## Author’s Note

The preregistration for Experiment 2, the analysis scripts, and the data (Experiments 1–2) are available at Open Science Framework (osf.io/6q7mh).

## Data Availability Statement

The datasets presented in this study can be found in online repositories. The names of the repository/repositories and accession number(s) can be found below: Open Science Framework (osf.io/6q7mh).

## Ethics Statement

The studies involving human participants were reviewed and approved by Office of Research Ethics, Waseda University, and die Ethikkommission der Deutschen Gesellschaft für Sprachwissenschaft. The patients/participants provided their written informed consent to participate in this study.

## Author Contributions

AI performed experiment design, data collection, data analysis, data interpretation, write-up (original draft). HS performed experiment design, data interpretation, and write-up (review). Both authors contributed to the article and approved the submitted version.

## Conflict of Interest

The authors declare that the research was conducted in the absence of any commercial or financial relationships that could be construed as a potential conflict of interest.

## Footnotes

1 Participants in Experiment 1 additionally performed a lexical decision test for another study that is not reported here.

2 Considering the claim that a growth curve analysis may produce a high rate of false positives due to over-fitting of the model and auto-correlation in eye movement data (Huang and Snedeker, 2018), we also analyzed the data using ANOVA (ezANOVA in ez package) and *t*-tests in the single time window from -800 to 200 ms. For this analysis, we transformed the fixation probabilities in this entire time window into log odds using the empirical logit function (Barr, 2008) and evaluated the log-transformed fixation probability as predicted by condition. The ANOVA showed a significant effect of condition, *F*(1.5, 85.0) = 228.4, MSE = 5.1, *p* < 0.001 (Greenhouse–Geisser correction). The follow-up paired *t*-tests showed a significant difference between the target and unrelated conditions, *t*(56) = 17.2, *p* < 0.001, as well as a significant difference between the orthographic and unrelated conditions, *t*(56) = 2.5, *p* = 0.02. The phonological condition did not differ from the unrelated condition, *t*(56) = 0.3, *p* = 0.8.

3 The ANOVA and *t*-tests equivalent to Experiment 1 found consistent results. The ANOVA found a significant effect of condition, *F*(1.7, 95.8) = 181.1, MSE = 3.7, *p* < 0.001 (Greenhouse–Geisser correction). The follow-up *t*-tests showed a significant difference between the target and unrelated conditions, *t*(55) = 15.1, *p* < 0.001, but no significant differences between the orthographic and unrelated conditions, *t*(55) = −0.29, *p* = 0.7, or between the phonological and unrelated conditions, *t*(55) = 1.1, *p* = 0.3.

## Appendix

Critical sentences with approximate English translations and critical objects for each condition. Target words are in bold. Cloze probabilities for target words are shown after each sentence in brackets.

**Table:**

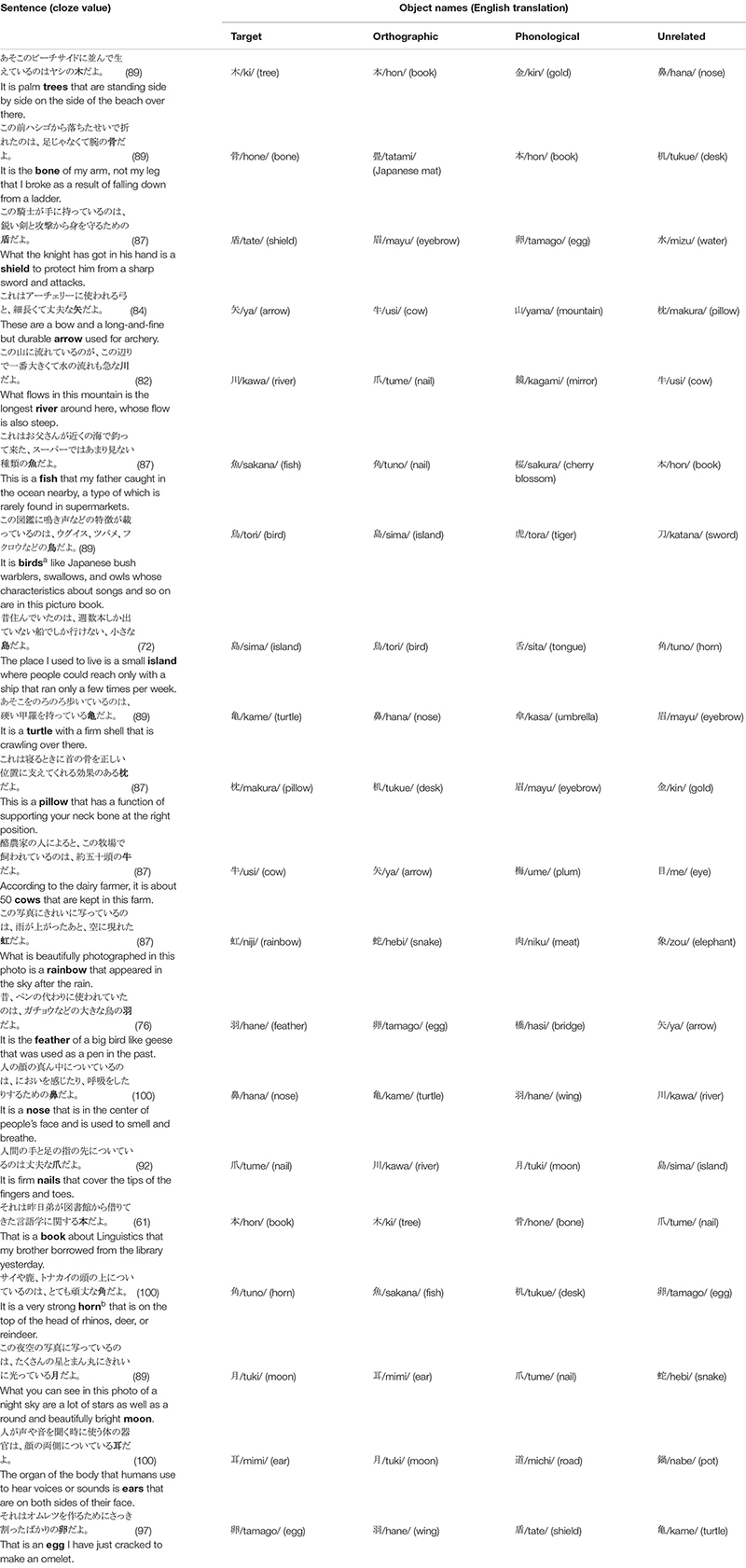

*^*a*^Plural is not marked in Japanese. ^*b*^A horn and antlers are both translated as /tuno/in Japanese.*
